# On the relationship between personal experience, affect and risk perception: The case of climate change

**DOI:** 10.1002/ejsp.2008

**Published:** 2014-07-05

**Authors:** Sander van der Linden

**Affiliations:** 1 Yale Project on Climate Change Communication, School of Forestry and Environmental Studies, Yale UniversityNew Haven, USA; 2 Department of Geography and the Environment and Grantham Research Institute, London School of Economics and Political Science (LSE)London, UK

## Abstract

Examining the conceptual relationship between personal experience, affect, and risk perception is crucial in improving our understanding of how emotional and cognitive process mechanisms shape public perceptions of climate change. This study is the first to investigate the interrelated nature of these variables by contrasting three prominent social-psychological theories. In the first model, affect is viewed as a fast and associative information processing heuristic that guides perceptions of risk. In the second model, affect is seen as flowing from cognitive appraisals (i.e., affect is thought of as a post-cognitive process). Lastly, a third, dual-process model is advanced that integrates aspects from both theoretical perspectives. Four structural equation models were tested on a national sample (N = 808) of British respondents. Results initially provide support for the “cognitive” model, where personal experience with extreme weather is best conceptualized as a predictor of climate change risk perception and, in turn, risk perception a predictor of affect. Yet, closer examination strongly indicates that at the same time, risk perception and affect reciprocally influence each other in a stable feedback system. It is therefore concluded that both theoretical claims are valid and that a dual-process perspective provides a superior fit to the data. Implications for theory and risk communication are discussed. © 2014 The Authors. *European Journal of Social Psychology* published by John Wiley & Sons, Ltd.

A “risk” is not something that exists “out there”, independent of our minds and culture (Slovic, 1992, p. 119). Indeed, unlike a physical threat or danger, the human notion of risk is a mental construct (Sjöberg, 1979), it cannot be sensed—it is only perceived (Fischhoff, Slovic, & Lichtenstein, 1983). Climate change is mostly a statistical concept, referring to the average long-term variability in the earth's climate (Solomon et al., 2007), and as such, it cannot be experienced directly (Swim et al., 2011). Compared with many other hazards, the threat of climate change is therefore relatively unique: not only because of its scope and breadth (Breakwell, 2010) but also in the sense that it is not directly “situated” in our daily environment (Helgeson, van der Linden, & Chabay, 2012).

Nevertheless, an increasing amount of research has shown that people can (to some extent) accurately detect changes in their local climate and relate this perceptual experience to climate change (e.g., Akerlof, Maibach, Fitzgerald, Cedeno, & Neuman, 2013; Howe et al., 2013; Joireman, Truelove, & Duell, 2010). Moreover, the rising incidence rate of extreme weather events is now increasingly being associated with climate change (Coumou & Rahmstorf, 2012). In fact, a number of studies have indicated that personal experience with extreme weather events is a significant predictor of climate change risk perceptions (e.g., Akerlof et al., 2013; Brody, Zahran, Vedlitz, & Grover, 2008; Krosnick, Holbrook, Lowe, & Visser, 2006; Spence, Poortinga, Butler, & Pidgeon, 2011).

Personal experience also plays a key role in affective processing, as affective responses are essentially formed through learning and experience (Damasio, 1994). An “*affective*” response is usually defined as a fast, associative, and automatic reaction that guides information processing and judgment (Zajonc, 1980). It is often described as a faint whisper of emotion, defined specifically as a positive (like) or negative (dislike) evaluative feeling toward a stimulus that can occur both consciously and unconsciously (Slovic, Finucane, Peters, & MacGregor, 2004). Risk perception often concerns future events (Sjöberg, 2000) and because affective evaluations of future risks largely depend on the vividness with which negative consequences can be represented mentally (Damasio, 1994; Weber, 2006), it logically follows that personal experience with the impacts of climate change and affective processing are closely interrelated (Marx et al., 2007). However, it is not only direct personal experience and affective processing that go hand in hand. In fact, it is often proposed that any perception (including risk) inevitably contains some affect (Zajonc, 1980). Indeed, negative affect has shown to be an important predictor of climate change risk perceptions (e.g., Leiserowitz, 2006; Sundblad, Biel, & Gärling, 2007).

Taken together, these research findings raise a number of important and unresolved questions about the relationship between personal experience, affect, and risk perception: does personal experience with extreme weather predict affective judgments? And in turn, do these affective judgments guide public risk perceptions of climate change? Or does personal experience predict risk perception, and in turn, does risk perception predict affect? Alternatively, is it possible that personal experience predicts risk perception and that risk perception and affect simultaneously and reciprocally influence each other?

In short, past research has failed to address the cognition-emotion dilemma in the context of climate change. Doing so is important because more effective public engagement with climate change necessitates risk communication strategies that can better take into account the way in which cognitive and experiential processes shape and influence public perceptions of climate change (Marx et al., 2007). As I will illustrate in the next section, the conceptual relationship between personal experience, affect, and risk perceptions of climate change can be represented within the frame of three competing social-psychological theories.

## The Present Study

### Model 1: Affect as an Information Processing Heuristic

Much research on affective processing assumes (either implicitly or explicitly) that affect is a *driver* of risk perception (rather than vice versa). For example, Zajonc (1980) unmistakably states that “affective judgments are fairly independent of, and precede in time, perceptual and cognitive operations” (p.1). In a similar vein, Slovic, Finucane, Peters and MacGregor (2007) assume that “the affect heuristic *guides* perceptions of risk” (p. 1343). The notion that people rely on affective cues when making risk judgments is pervasive and based on a substantial amount of experimental as well as clinical research (e.g., Damasio, 1994; Dohle, Keller, & Siegrist, 2010; Finucane, Alhakami, Slovic, & Johnson, 2000; Johnson & Tverksy, 1983; LeDoux, 1996; Schwartz & Clore, 1983; Zajonc, 1984). The underlying idea is that fast affective judgments are an evolutionarily adaptive and efficient way of processing information, especially when navigating in a complex, uncertain, and dangerous world (Slovic et al., 2007).

**Figure 1:**
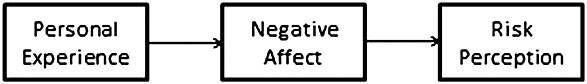
Hypothesized causal flow model 1 (the “affective” model)

Until now (and with good reason), *affect* has mostly been conceptualized as a *predictor* of climate change risk perceptions (e.g., Leiserowitz, 2006; Smith & Leiserowitz, 2012; Sundblad et al., 2007). In line with this view, the hypothesized causal relationship is expected to be unidirectional, where more personal experience with extreme weather leads to the development of negative affective evaluations, which in turn cultivate higher risk perceptions of climate change. A description of the “affective” model is presented in Figure 1.

### Model 2: Affect as a Post-Cognitive Process

Other recent experimental research has questioned the extent to which affect can be seen as an associative construct (e.g., Townsend, Spence, & Knowles, 2013) and suggested appraisal theory as a means of understanding the role of emotion in risk perception (e.g., Keller et al., 2012). In fact, diametrically opposed to Zajonc (1980, 1984), Lazarus (1981, 1984) proposed that cognitive processes mediate the relation between environmental situations and specific emotional reactions (i.e., emotions are seen as a post-cognitive process). In order to explain how different people can experience different emotions in identical situations, Lazarus argued that events are first appraised (i.e., perceived and interpreted) in terms of their personal meaning (i.e., how they relate to an individual's past experiences, personal values, and overall well-being). Like information processing theories, “appraisal theory” has marshaled substantial empirical support (e.g., Roseman, 1984; Scherer, Schorr, & Johnstone, 2001; Siemer, Mauss, & Gross, 2007). In fact, Loewenstein, Weber, Hsee, and Welch (2001) state “few would question that cognitive evaluations give rise to affective responses” (p. 271).

**Figure 2:**
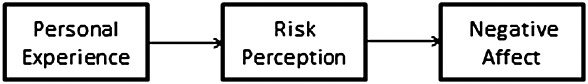
Hypothesized causal flow model 2 (the “cognitive” model)

In support of this view, Kobbeltved, Brun, Johnsen, and Eid (2005) reported in their (cross-lagged) panel study that “it is unlikely that our subjects allowed their affective impressions to guide their risk judgments” (p. 431). Instead, the authors note that over time, risk judgments gave rise to negative emotions but not vice versa. Thus, in line with this paradigm (and in contrast to the affect-heuristic hypothesis), an alternative hypothesized causal relationship is one where more personal experience with extreme weather events leads to higher risk perceptions, which in turn, create (more) negative affective evaluations of climate change. A description of the “cognitive” model is presented in Figure 2.

### Model 3: A Dual-Processing Perspective—The Case of Climate Change

It is important to recognize, however, that both theoretical perspectives have validity and are not mutually exclusive. For example, although it is now obvious that certain older, subcortical structures in the brain can receive (sensory) information independently of the neocortical structures related to cognition (LeDoux, 1996), when an individual is faced with extreme weather he or she is likely to activate *both* affective processing (e.g., danger) and cognitive information about its ontological category (e.g., tornado and climate change). Indeed, humans perceive risk in two fundamental ways, namely, in a cognitive-analytical and experiential-affective manner (Slovic & Peters, 2006). Yet, there is an increasing consensus that the way in which affect functions in relation to cognition is strongly dependent on the context (Lai, Hagoort, & Casasanto, 2012).

**Figure 3:**
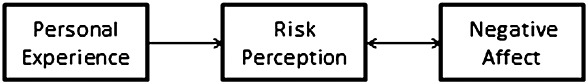
Hypothesized causal flow model 3 (the “dual-process” model)

In fact, Loewenstein et al. (2001) suggest that when cognitive and emotional responses diverge, reactions are more likely to be guided by affect. Yet, climate change is an evolutionarily novel risk that does *not* represent a clearly observable physical danger, and thus, there is no environmental cue present to automatically trigger an affective, fear-based fight-flight type response that bypasses cognition completely (Griskevicius, Cantu, & van Vugt, 2012; Weber, 2006). Instead, it is much more likely that when someone personally experiences the likely consequences of climate change (i.e., extreme weather), the individual must first cognitively and causally attribute his or her perceptual experience to climate change (Weber, 2010; Helgeson et al., 2012). At the same time, when this link has been made salient, it is equally likely that negative affective reactions guide and exert a strong influence over risk perceptions. This idea is consistent with neurobiological evidence, which suggests that affect can influence cognition and cognition can influence affect (LeDoux, 1989). To this extent, appraisal studies have started to conceptualize the link between emotion and cognition as bi-directional (e.g., Nerb & Spada, 2001), and research is now steadily moving toward a dual-process perspective—where the interplay between emotion and cognition is increasingly recognized (e.g., Clore & Ortony, 2000; Gray, 2004; Loewenstein et al., 2001; Pessoa, 2008; Phelps, 2006). Consistent with this view, the current study presents a dual-process model (Figure 3), where more personal experience with extreme weather is expected to predict higher risk perceptions, and while higher risk perceptions in turn predict affect, negative affective evaluations are expected to simultaneously influence and drive higher risk perceptions of climate change. The purpose of the present research is to empirically evaluate the plausibility of all three hypothesized model structures.

## Method

### Sample and Participants

The dataset consists of a national sample (*N* = 808) of the population of the United Kingdom. Because the UK has a high degree of Internet users (about 77% of the population), the survey was conducted online via a survey sampling company. A nationally balanced quota sample^1^ (based on gender, age, and region) was obtained from a large mixed panel of people who were willing to participate in Web-based research for a small (symbolic) reward. Multi-stage randomization was used to select participants from the panel. The sample comprised 50% male and 50% female respondents. The age of participants ranged between 18 and 65, with a modal age bracket of 35–44.

### Materials and Procedure

Because the survey is part of a larger study that aims to explore and investigate a wide range of perceptions, attitudes, and behaviors related to climate change, only relevant constructs and results are reported here. The survey instrument was designed with input from a panel of three academic and professional experts. Furthermore, to ensure that the survey questions and response categories were clear and unambiguous, a pilot study was conducted at the behavioral research lab of the London School of Economics with a focus group of (*N* = 15) members of the general public. Results of the pilot study were used to refine the questionnaire. The survey was administered online in October 2012 and took about 15–20 min to complete.

### Measures

#### Risk Perception

A total of eight measures were used to assess risk perception. Drawing on standardized items developed by O'Connor, Bord, and Fisher (1999) and Leiserowitz (2006), all constructs were measured on seven-point (unipolar) Likert-type scales and covered both spatial and temporal risk dimensions. The first two questions asked respondents to judge how *likely* they think it is that they will personally experience threats to their overall well-being as a result of climate change. The same was asked for society as a whole. Three questions asked respondents to evaluate how *serious* of a threat they think climate change is to the natural environment, the UK, and to them personally. Respondents were also asked how serious they would rate current impacts around the world and how *concerned* they are about climate change in general. For analysis, a *holistic* measure of risk perception was created (α = .96).

#### Generalized Affect

Peters and Slovic (2007) tested and cross-compared numerous ways of measuring self-reported affect (e.g., through imagery and discrete emotions). The authors found that among all measures, broadly valenced or “holistic” affective evaluations proved to be most reliable. Accordingly, generalized affect was measured with three broadly evaluative bipolar adjective scales, for example, “I feel that climate change is” (very unpleasant–pleasant, unfavorable–favorable, and negative–positive). A reliable scale was obtained (α = .85).

#### Personal Experience with Extreme Weather Events

Because rising sea levels are one of the most probable consequences of climate change (Solomon et al., 2007), previous research on extreme weather has predominantly focused on flooding events (e.g., Spence et al., 2011; Whitmarsh, 2008). Yet, to account for a potentially wider range of personal experiences with extreme weather, two separate questions were used. Respondents were first asked to recall how often in the last 5 years they had experienced (i) flooding and (ii) other extreme weather events (e.g., severe heat waves, droughts, and freak storms) while in the UK. An affirmative response to either question counted toward personal experience. Responses were combined and dichotomized to form an index describing personal experience (0 = no experience, 1 = experience).

#### Cause Knowledge

Knowledge about the causes of climate change is used in the current study as an auxiliary “instrument” (which is further discussed in the following section). Measures that try to assess subjective or “self-reported knowledge” with a single item tend to be unreliable (Roser-Renouf & Nisbet, 2008). Therefore, cause knowledge was assessed with 13 items that were presented in random order (seven of which were correct statements and six were incorrect). The correctness of all statements was based on expert reports (e.g., Intergovernmental Panel on Climate Change (IPCC)) and checked by two climate scientists. Respondents were asked to what extent each item (e.g., burning fossil fuels) contributes to climate change (i.e., major, minor, or no contribution). Following a method developed by Leiserowitz, Smith, and Marlon (2010), responses were scored as either right (1) or wrong (0) and indexed (0–13) based on the number of *correct* answers, where more correct answers reflect higher knowledge. A reliable scale was obtained for cause knowledge (α = .90).

## Results

### Overview of Statistical Analyses

All analyses were performed using a latent variable structural equation modeling (SEM) approach (Bollen, 2005; Ullman & Bentler, 2013).

#### Recursive versus Nonrecursive Structural Equation Models

The hypothesized relationships of the “cognitive” and “affective” models are estimated with a standard *recursive* (i.e., unidirectional) structural equation approach. However, the dual-process model is estimated using a *nonrecursive* (i.e., a feedback or bi-directional) structural equation model. Nonrecursive structural equation models are more complicated in the sense that both variables (here risk perception and affect) are thought to simultaneously “cause” or influence each other. The problem of simultaneous causation is often addressed with *cross-lagged* models, yet, this is certainly not always appropriate because (i) the assumed “lag” between cause and effect is usually unknown (Wong & Law, 1999) and (ii) cause does *not* have to precede effect in time (e.g., it is entirely reasonable that the perception of risk and the experience of negative affect occur simultaneously or at least in short succession).

In fact, nonrecursive structural equation models estimated on cross-sectional data implicitly make a so-called functional equilibrium assumption (Schaubroeck, 1990) in order to obtain unbiased regression estimates (Kenny, 1979). The assumption implies that if a causal feedback loop has not fully materialized or stabilized yet, cross-sectional data would not be able to substantiate a synchronous bi-directional relationship. Another important (and often neglected) assumption of nonrecursive structural equation models is that they need to be empirically identified (Kenny, 1979), which is only truly the case when each endogenous variable in the model has its own instrument (i.e., an exogenous variable that influences Y_1 (affect)_ but not Y_2 (risk)_ and vice versa). In other words, if two variables are believed to cause each other, a third variable (the instrument) is needed to partition the variance into endogenous and exogenous components.

For example, in the current study, a valid instrument would be a variable that is significantly correlated with *affect* but not (or only marginally) to risk perception. The instrumental variable approach to causal inference is a powerful but underutilized tool in psychology (Bollen, 2012). In the current study, knowledge about the *causes* of climate change is introduced as an instrument. It is argued here that cause knowledge is a valid instrument because it is both theoretically and empirically related to affect (Y_1_) but only marginally to risk perception (Y_2_). To illustrate, in contrast to knowledge about the *impacts* of climate change (which are often vivid descriptions of a risk event), knowledge about the *physical causes* is expected to share a much weaker theoretical link with risk perception. Particularly, because regardless of whether climate change is seen as a natural or human-caused threat, it remains a risk. Yet, the fact that it is human-caused is likely to trigger strong negative emotional reactions. Indeed, the main perceptual difference between natural and man-made risks often lies in negative affective reactions toward the inflicting agent (Böhm, 2003; Brun, 1992).

#### Assessment of Model Fit

Model fit is assessed using a range of goodness of fit statistics.^2^ The first test statistic reported is the χ^2^ (Chi-square)—for which lower values indicate better fit. The Comparative Fit Index and the Tucker Lewis Index are comparative (relative) fit indices where a cutoff value of 0.95 indicates good fit and >0.95 excellent fit. The root mean square error approximation (RMSEA) is an absolute fit index that measures lack of fit per degree of freedom, where cutoff values between 0.05 and 0.10 indicate reasonable fit and values <0.05 excellent fit. Nested models (i.e., when a model is simply a less restricted version of the other) are assessed with a Chi-square difference test. Nonnested models are evaluated with parsimony fit indices such as the Akaike Information Criterion and the Bayesian Information Criterion (BIC) where again, lower values indicate better fit. More specifically, a difference of 10 or more provides *very strong* evidence that the model with the more negative values has better fit (Raftery, 1995).

### Validating the Measurement Model

With regard to the measurement model, it is important to first establish sufficient content and construct validity of the measures. As discussed, most of the items used for risk perception and affect are relatively standardized, and their usefulness has been established in prior research. Nonetheless, convergent and discriminant validity of the items is assessed using the multitrait-multimethod approach (Campbell & Fiske, 1959) and confirmatory factor analysis—for which results are presented in Tables 1 and 2.

**Table 1:** Factor loadings and scale reliabilities of measurement items

Risk perception measures	Factor loadings	Cronbach's α
Risk perception index		.96
Risk perception item 1	0.90	
Risk perception item 2	0.72	
Risk perception item 3	0.86	
Risk perception item 4	0.91	
Risk perception item 5	0.92	
Risk perception item 6	0.86	
Risk perception item 7	0.92	
Risk perception item 8	0.76	
Generalized affect index		.85
Affect item 1	0.89	
Affect item 2	0.91	
Affect item 3	0.70	

*Note*: All factor loadings are significant at *p* < .001. Squared factor loadings (**λ**^**2**^) indicate the amount of variance that the item explains in the latent construct.

**Table 2:** Descriptive statistics and intercorrelations

*N* = 808	Risk perception	Generalized affect	Cause knowledge	Personal experience	Mean	Standard deviation
Risk perception	(0.96)				4.83	1.36
Generalized affect	0.54***	(0.86)			5.33	1.20
Cause knowledge	0.09*	0.22***	(0.90)		6.24	1.92
Personal experience	0.22***	0.08*	−0.02	(1.0)	N.A	N.A

*Note*: Personal experience is a dichotomous variable (1 = experience, 0 = no experience). Measures are coded so that higher scores reflect more of the construct. Reliability scores (α) are provided in parentheses along the main diagonal.

N.A, not applicable.

** *p* < .05;

**p < .01;

*** *p* < .001.

The factor loadings are provided in Table 1 and indicate that all risk perception and affect items load highly on their respective factors, illustrating good *convergent* validity (Farrell & Rudd, 2009). In addition, the multitrait-multimethod approach suggests that an item should correlate more strongly with all items of the same construct than with measures of other constructs (Campbell & Fiske, 1959). For example, the items measuring risk perception should correlate more strongly with each other than with the items measuring affect. In other words, the *inter*correlation between risk perception and affect (*r* = .54) should not exceed the value of Cronbach's alpha (α = .96) given that α is an *intra*-class correlation coefficient. This is clearly the case, as Table 2 illustrates. All intercorrelations are well below their mean scale reliabilities, indicating sufficient *discriminant* validity^3^ between the measures (Farrell & Rudd, 2009).

### The “Affective” versus “Cognitive” Model

**Figure 4:**
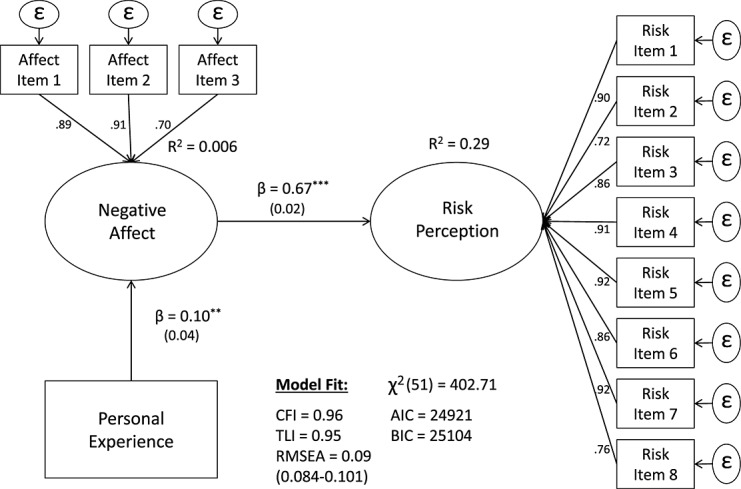
*Affect as an information processing heuristic* (the “affective” model). Entries are standardized beta coefficients. Only main results are depicted for easy of interpretation, ^*^p < 0.05;^**^p < 0.01;^***^p < 0.001.

**Figure 5:**
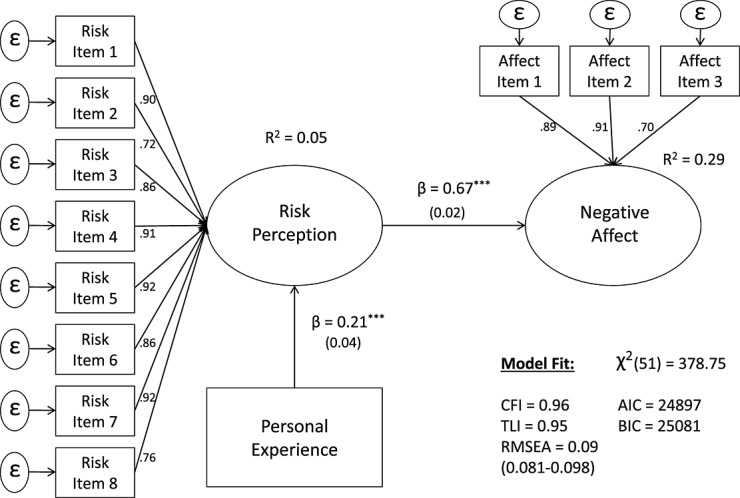
*Affect as a post-cognitive process* (the “cognitive” model). Entries are standardized beta coefficients. Only main results are depicted for easy of interpretation, ^*^p < 0.05;^**^p < 0.01;^***^p < 0.001.

The structural equation models were estimated using stata's SEM package, StataCorp, College Station, Texas, USA.^4^, and results are presented in Figures 4 and 5. The “affective” model is presented first in Figure 4, where affect is conceptualized as an antecedent of risk perception (i.e., cognition). Results indicate that both paths, from personal experience to negative affect (β = .10, *SE* = 0.04, *p* < .01) and from negative affect to risk perception (β = .67, *SE* = 0.02, *p* < 0.001), are significant. Yet, while personal experience with extreme weather seems to explain very little to no variance in negative affect (*R^2^* = 0.006), negative affect explains a substantial amount of variance in risk perceptions of climate change (*R^2^* = 0.29).

In contrast, the “cognitive” model, where risk perception is conceptualized as an antecedent of affect, seems to be better specified in terms of predictive ability—given the magnitude of the personal experience to risk perception path (nearly double) and improved *R^2^* = 0.05 (Figure 5). Moreover, a detailed inspection of the direct and indirect effects (followed by a mediation test) indicates that the impact of personal experience on affect is in fact fully mediated by risk perception (*z* = 5.67, *SE* = 0.05, *p* < .001).

In terms of overall model fit, the “cognitive” model (Figure 5) also has a lower χ^2^ (51) = 378.75, *p* < .001 than the “affective” model (Figure 4) χ^2^ (51) = 402.71, *p* < .001—which is preferable. Yet, because these models are *nonnested* (i.e., one model is not simply a more restricted version of the other), the parsimony fit indices are also compared to further assess model fit. The differences in the Akaike Information Criterion Δ(*AIK*) = −24 and the Δ*BIC* = −23 are strongly in favor of the “cognitive” model. Although both models seem to have a reasonable *absolute* fit (*RMSEA* = 0.09), the “cognitive” model is clearly superior in terms of its *R^2^* and *relative fit*—where personal experience is best conceptualized as a predictor of risk perception and, in turn, risk perception a predictor of affect (i.e., affect is *post-cognitive*).

### A Dual-Process Model of Risk Perception and Affect

The aforementioned *recursive* (i.e., unidirectional) models provide few clues about the potentially interdependent and reciprocal relationship between risk perception and affect. Therefore, a logical next step is to compare the recursive “cognitive” model to a dual-process model where risk perception and affect simultaneously influence each other. Yet, in order to estimate a nonrecursive (feedback) model, each of the endogenous variables (risk perception and affect) requires an exogenous instrument. As hypothesized in the introduction, without any (conscious) cognitive attribution of the risk event, personal experience with extreme weather seems to have little theoretical connection with affectivity toward climate change. The data support this hypothesis, given that the effect of personal experience on affect is fully mediated by risk perception. In fact, as Table 2 indicates, while personal experience is significantly correlated to risk perception (*r* = .22, *p* < .001), it is only marginally related to affect (*r* = .08, *p* < .05). Thus, personal experience naturally functions as a viable instrument for risk perception.

The second instrument employed is knowledge about the *causes* of climate change. It was hypothesized that knowledge about the *physical causes* of climate change would share a weak theoretical relationship with risk perception but not with affect. This notion is also supported, as Table 2 clearly shows that cause knowledge is significantly related to affect (*r* = .22, *p* < .001) but only marginally to risk perception (*r* = .09, *p* < .05). Moreover, the effect of cause knowledge on risk perception is fully mediated by affect (*z* = 6.10, *SE* = 0.01, *p* < .001). One potential concern to keep in mind is that if the instruments have differential effects on their corresponding endogenous variables, the variable with the weaker instrument will have a greater disturbance term (Wong & Law, 1999).

**Figure 6:**
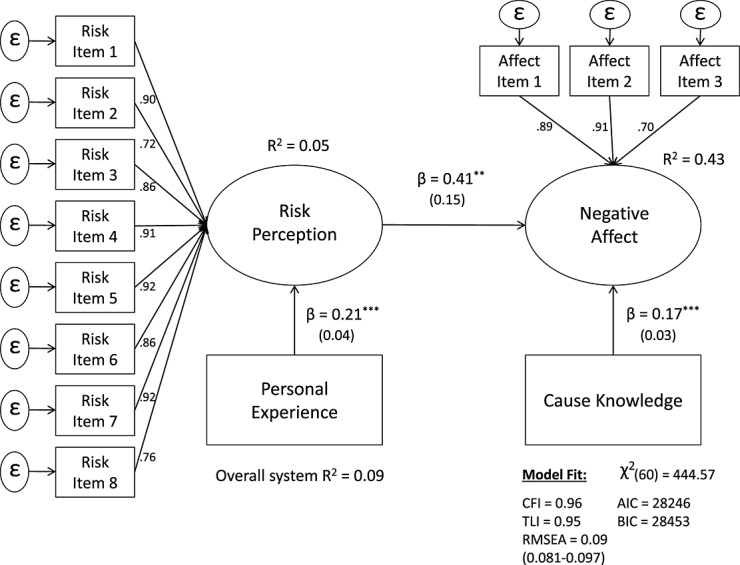
*Recursive structural equation model*. Entries are standardized beta coefficients. Endogenous variables covary freely, ^*^p < 0.05;^**^p < 0.01;^***^p < 0.001.

**Figure 7:**
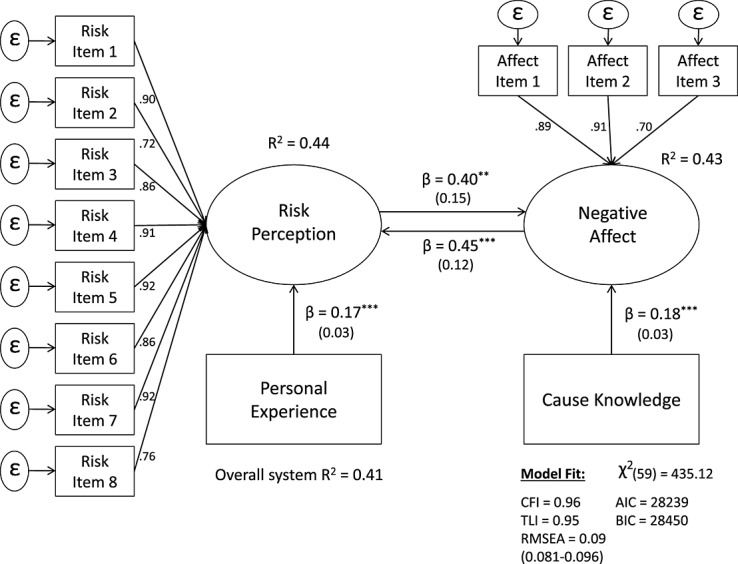
*Nonrecursive (dual-process) structural equation model*. Entries are standardized beta coefficients. Endogenous variables covary freely. *R*^2^'s are Bentler–Raykov squared multiple correlations (Bentler & Raykov, 2000), ^*^p < 0.05;^**^p < 0.01;^***^p < 0.001.

The recursive “cognitive” model is estimated first (Figure 6) and subsequently compared with the reciprocal (nonrecursive) model (Figure 7).

A few observations are immediately evident. First, to mitigate concerns about the instruments, their standardized effect on each of the corresponding endogenous variables appears to be equal (β = .17, *SE* = 0.03, *p* < .001 vs. β = .18, *SE* = 0.03, *p* < .001). Second, both of the path coefficients, from risk perception to affect (β = .40, *SE* = 0.15, *p* < .01) and from affect to risk perception (β = .45, *SE* = 0.12, *p* < .001), are significant. Third, the overall explained variance (*R^2^* = 0.41) of the reciprocal (dual-process) model is substantially higher than that of the unidirectional model (*R^2^* = 0.09).

In terms of model fit, because the two models are nested (i.e., one is a less restricted version of the other), a Chi-square difference test was performed. The difference in Δχ^2^ (1) = 9.45 between the two models is significant (*p* < .01), indicating that the nonrecursive (i.e., reciprocal) model fits the observed variance-covariance matrix better than the unidirectional model. The difference in the parsimony fit indices, Δ*AIC* (−6) and Δ*BIC* (−3) are equally in favor of the reciprocal model (Figure 7). To further examine the plausibility of the bi-directional relationship, the path coefficients between risk perception and affect were constrained to be equal (this adds a degree of freedom as now only one path needs to be estimated, causing the model to be overidentified). The difference in Δχ^2^ (1) = 2.19 between the constrained and freely estimated model is not significant, indicating that the two path coefficients are equal. Lastly, the functional equilibrium assumption was tested. A stable model returns an index eigenvalue between 0 and 1. Since the returned value was 0.41, the nonrecursive model satisfied the stability condition (Bentler & Freeman, 1983). Overall, these findings strongly point to the conclusion that a nonrecursive model (where risk perception and affect reciprocally influence each other) provides a better and more plausible fit to the data than a recursive (i.e., unidirectional) model.

## Discussion

### A Causal Tale: Personal Experience with Extreme Weather, Negative Affective Evaluations, and Risk Perceptions of Climate Change

The current study set out to explore the complex and intricate causal relationship between personal experience with extreme weather events, negative affect, and risk perceptions of climate change. Using a SEM approach, three competing theories were contrasted, namely, (i) the “affective” model (where affect is seen as information processing heuristic), (ii) the “cognitive” model (where affect is seen as a post-cognitive process), and (iii) a “*dual-process*” model that integrates aspects from both theoretical perspectives. Results initially provided support for the post-cognitive specification, where personal experience predicts risk perception and, in turn, risk perception predicts (negative) affect. However, further examination of the relationship between risk perception and affect revealed that while personal experience is indeed best conceptualized as a predictor of risk perception, a mutually reinforcing and reciprocal relationship between affect and risk perception provides a significantly better fit to the data than a unidirectional model.

### Toward a Dual-Processing Perspective

Previous research has noted that because climate change (as an object) cannot be experienced directly, it fails to activate a primal affective/associative risk response (Weber, 2006). In fact, it has been suggested that in order for an individual to develop negative affectivity toward climate change, that individual must first cognitively attribute personal experience with extreme weather to climate change (Helgeson et al., 2012; Weber, 2010). Results of the current study are congruent with this post-cognitive interpretation, as the effect of personal experience with extreme weather events is fully mediated by risk perception. These results strongly suggest that people first perceive, interpret, and appraise risk events in terms of their personal meaning and relevance (at least in the context of climate change). As such, these results are consistent with appraisal theory (e.g., Böhm, 2003; Lazarus, 1984; Nerb & Spada, 2001; Zaalberg, Midden, Meijnders, & McCalley, 2009), other risk perception studies (e.g., Kobbeltved et al., 2005; Yang & Kahlor, 2013), and recent research that has provided evidence for a reciprocal relationship between personal experience and belief certainty that climate change is happening (Myers et al., 2012).

It is important to note that these results by no means negate the role of affect as a fast and associative response heuristic that guides information processing and risk judgments, as suggested by Zajonc (1980) and Slovic et al. (2004). In fact, it is very likely that extreme weather events trigger biologically hard-wired affective reactions (e.g., an impending hurricane is likely to stir up negative emotional reactions). Yet, the experienced affect is geared toward the risk object (i.e., the hurricane) and does not necessarily influence perceptions of climate change (unless a conscious link between the extreme weather event and climate change is made salient). However, as this study shows, once this link is established, negative affective reactions appear to be a strong determinant of climate change risk perceptions, where risk perception and affect mutually reinforce each other in a stable feedback system. This is consistent with prior research that has shown that affective judgments of climate change predict cognitive risk perceptions and vice versa (Sundblad et al., 2007).

In conclusion, the current study strongly suggests that (at least in the context of climate change) both theoretical perspectives hold true: affect can be seen as a post-cognitive process as well as an information processing heuristic that guides perceptions of risk. These findings are entirely consistent with research that has pointed out the interrelated neurological connections between the brain's subcortical and neocortical structures (e.g., LeDoux, 1995; Pessoa, 2008) and provide further empirical evidence for the validity of dual-processing theories that highlight the interplay between cognition and emotion (e.g., Loewenstein et al., 2001; Sloman, 1996; van der Linden, 2014).

### Implications for Risk Communication

The practical value of this research is evident in that risk perception, and the experiential system in general has been implicated as an important determinant of actions to help reduce climate change (e.g., O'Connor et al., 1999; Leiserowitz, 2006; Marx et al., 2007; Semenza et al., 2008; Spence et al., 2011). Results of this study suggest that in order to design effective social-psychological interventions, risk communication messages should take into account the interrelated nature of personal experience, affect, and risk perception and the way in which these variables shape perceptions of and beliefs about climate change. Indeed, the interactive engagement of both cognitive and emotional processing mechanisms is key to fostering more public involvement with climate change.

For example, based on the findings of this study and others (e.g., Myers et al., 2012; Capstick & Pidgeon, 2014), risk communication campaigns should try to emphasize the association between more frequent extreme weather events and climate change. Particularly, because once this link is made salient, a mutually reinforcing relationship between risk perception and affect is established. In addition, results suggest that negative affective evaluations can also be elicited by improving the public's (cognitive) knowledge of the human causes of climate change. In turn, negative affect is then likely to further guide information-seeking behavior (e.g., Yang & Kahlor, 2013). Facilitating the cognition-emotion link is congruent with recent research on climate change communication, which equally suggests that appealing to *both* cognitive and affective processing mechanisms is likely to be a more successful approach (van der Linden, 2014).

### Some Final Thoughts on Cognition, Emotion, Causality, and Future Research

It is well known that the cognition versus emotion debate in psychology is held back by semantics (Kleinginna & Kleinginna, 1985; LeDoux, 1995). In other words, the relationship between cognition and affect, to some extent, depends on how we choose to define these concepts. I should therefore note that the risk perception measures used in this study might not be entirely cognitive, and similarly, measures of affect might not be judged as purely emotional either, depending on the reader's personal definition of these concepts. Yet, they do not need to be, the standardized measures used in the current study display sufficient convergent and discriminant validity. Moreover, the purpose of this paper has been to illustrate how these psychological constructs function in relation to each other in the context of climate change. In line with the views expressed by Lai et al. (2012), future research is advised to move away from discussing semantics and instead focus on exploring the functional relationships between cognitive and experiential constructs in specific contexts.

In addition, I would like to offer a final note on the term “causality” and how it relates to SEM. As Pearl (2012) summarized, the critical reader might ask, “*structural equation modeling cannot prove causation*” *so how can it yield results that have a causal interpretation*? (p. 1). While it is true that no statistical method can in or by itself truly prove “causality,” the current study stresses that through a combination of theory and observation, parameters can certainly have a causal interpretation (Iacobucci, 2009; Pearl, 2012) and encourages the view that SEM should be seen as a tool to assess the *plausibility* of different hypothesized causal path relationships (based on how well such theoretical structures fit the observed variance-covariance matrix).

As Fabrigar, Porter, and Norris (2010) state,

If one model is found to be clearly superior to other models, a researcher might reasonably make the case that certain causal assumptions are more plausible than others for the given dataset (p. 222).

It is always good to keep in mind that depending on (i) one's philosophy of causality, (ii) the data at hand and (iii) the extent to which all the assumptions of a given statistical method are met, “it will not in general be indisputably clear that an experimental approach accords with all the criteria for causation better than a nonexperimental approach” (Bagozzi, 2010, p. 210).

Human social life is invariably complex and attaining a better understanding of causal relationships necessitates a methodology that is flexible and dynamic enough to model intricate behavioral systems. To this extent, SEM can help researchers offer tentative causal conclusions (Bullock, Harlow, & Mulaik, 1994; Fabrigar et al., 2010; Markus, 2010). The emphasis here is on the term *tentative*, and future research in this area could constructively build on the current study by using experimental techniques as well as panel data to further assess the causal structure between personal experience, risk perception, and affect. In particular, future studies could explore how these relationships are likely to function and behave over time as well as how they relate to intention and behavior.

## Notes

1 Quotas reflect the 2001 census data for the Great British population.

2 For detailed guidelines of goodness of fit statistics in structural equation modelling please see McDonald and Ho (2002) and Hu and Bentler (1999) for a good overview.

3 Similarly, the confirmatory factor analysis results can be used to prove that the shared variance (squared correlation) between each pair of constructs is lower than the extracted variance (the average of the squared factor loadings).

4 Because of some item nonresponse (approximately 10%), the model was estimated using a full information maximum likelihood procedure—which produces unbiased estimates and is highly recommended (Enders & Bandalos, 2001).
